# Endovascular Therapy in Acute Isolated Posterior Cerebral Artery Occlusion: Systematic Review and Meta-analysis

**DOI:** 10.1007/s00062-022-01221-7

**Published:** 2022-10-20

**Authors:** Ramy Abdelnaby, Khaled Ashraf Mohamed, Anas ELgenidy, Yousef Tarek Sonbol, Mahmoud Mostafa Bedewy, Aya Moustafa Aboutaleb, Khaled Tarek Dardeer, Hamed Amr Heikal, Hazem Maher Gawish, Omid Nikoubashman, Arno Reich, João Pinho

**Affiliations:** 1 Department of Neurology, University Hospital, University Hospital RWTH Aachen, Pauwelsstr. 30, 52074 Aachen, Germany; 2 Faculty of Medicine, Cairo University, Cairo, Egypt; 3 Faculty of Medicine, Zagazig University, Zagazig, Egypt; 4 Department of Diagnostic and Interventional Neuroradiology, University Hospital, RWTH Aachen University, Aachen, Germany

**Keywords:** Ischemic stroke, Stroke, Large vessel occlusion, Thrombectomy, Outcome

## Abstract

**Purpose:**

Patients with isolated posterior cerebral artery occlusion (iPCAO) represent up to 6% of all acute ischemic stroke patients. Acute revascularization therapies for these patients were not tested in randomized controlled trials. The aim of this study was to evaluate outcomes of iPCAO patients who undergo endovascular treatment (EVT).

**Methods:**

A systematic search of MEDLINE, Web of Science, CENTRAL, Scopus (inception—03/2022) was conducted for studies reporting 3‑month outcome, symptomatic intracranial hemorrhage (sICH) and/or successful recanalization in iPCAO patients who underwent EVT. Random effect meta-analyses for pooled proportions were calculated. Double-arm meta-analyses for comparison of outcomes of iPCAO patients treated with EVT with age-, sex- and NIHSS-matched iPCAO patients treated with best medical treatment only were performed.

**Results:**

Fifteen studies reporting a total of 461 iPCAO patients who underwent EVT were included. Excellent and favorable 3‑month outcome proportions were 36% (95% confidence interval, CI 20–51%) and 57% (95% CI 40–73%), respectively. The 3‑month mortality was 9% (95% CI 5–13), sICH occurred in 1% (95% CI 0–2%), successful recanalization was achieved in 79% (95% CI 71–86%). No significant differences in favorable and excellent 3‑month outcomes, 3‑month mortality and symptomatic intracerebral hemorrhage were found between the groups of patients who underwent EVT and the group of patients who received best medical treatment only.

**Conclusion:**

These results support the feasibility and safety of EVT in iPCAO, but do not show an outcome benefit with EVT compared to best medical treatment. Randomized trials are needed to evaluate treatment benefit of EVT in these patients.

**Supplementary Information:**

The online version of this article (10.1007/s00062-022-01221-7) contains supplementary material, which is available to authorized users.

## Introduction

Although the benefit of endovascular treatment (EVT) in acute ischemic stroke patients with anterior circulation large vessel occlusion is well established, several questions remain unresolved, namely the treatment of patients with posterior circulation large vessel occlusion and medium vessel occlusion [1, 2]. Patients with infarcts in the territory of the posterior cerebral artery (PCA) account for 5–6% of all acute ischemic stroke patients [3, 4]. Isolated PCA infarcts can cause of a wide range of neurological deficits including decreased vigilance, visual field defects, severe motor deficits and incapacitating neuropsychological deficits [5]. In a population-based stroke register conducted between 1998 and 2009, approximately 50% of all patients with first-ever isolated PCA infarcts, of whom the vast majority had not received any acute revascularization therapy, had moderate to major disabilities or had died 6 months after stroke [4]. The benefit of the currently available revascularization therapies in this subgroup of patients, i.e. intravenous thrombolysis (IVT) and EVT, was not systematically tested in randomized controlled trials. Nonetheless, the implementation in everyday clinical practice has gained acceptance in the era of acute reperfusion therapy. Several observational studies which analyzed patients with isolated posterior cerebral artery occlusion (iPCAO) who underwent EVT suggest that the procedure in these patients is safe and may lead to clinical benefit. However, it is unclear whether EVT when compared to best medical treatment is beneficial.

We performed a systematic review of the evidence describing outcomes of patients with iPCAO who underwent EVT. Additionally, we aimed to compare outcome in patients with iPCAO treated with EVT when compared to a matched population who underwent best medical treatment only.

## Material and Methods

We conducted a systematic review and meta-analysis according to the Preferred Reporting Items for Systematic Reviews and Meta-Analyses (PRISMA) guidelines [6]. The current study was not previously registered and there is no published study protocol. The PRISMA checklist can be found in the Supplementary Table 1.

### Search Strategy

We searched MEDLINE (PubMed), Web of Science, Cochrane Central Register of Controlled Trials (CENTRAL), Embase, and Scopus using a comprehensive search term to identify studies published until 30 March 2022, with no language restriction and no restriction of study design. Three authors (A.E, Y.T.S. and M.B.) searched the mentioned databases using the following search terms: thrombectomy* OR “endovascular treatment” OR “endovascular stroke treatment” OR embolectomy* OR “stent retriever” OR “stent-retriever” OR “aspiration” OR thrombol* OR fibrinol* OR rtPA OR rt-PA OR alteplase AND stroke OR ischem* OR ischaem* OR infarct* OR cerebrovascular AND “posterior cerebral artery” OR P1 OR P2 OR P3.

### Eligibility Criteria

We included studies which fulfilled the following criteria: full text published in peer-reviewed journals; report of acute ischemic stroke patients with iPCAO who underwent EVT; articles reporting any of change in severity of neurological deficits after treatment, short-term functional outcome, death, vessel reperfusion frequency, symptomatic intracranial hemorrhage (sICH). Exclusion criteria were: conference abstracts; animal studies; isolated case reports; studies which included only patients with simultaneous PCA and other intracranial artery occlusions; studies in which specific data for patients with iPCAO were not available; studies with possible overlapping study populations. In the case of studies with overlapping populations we included only the study with the largest population size. Four authors (A.E., Y.T.S., M.B., A.M.) independently screened the title and abstract of all articles, and articles of potential relevance were selected. The full text of these articles was reviewed for eligibility, and disagreements on eligibility were settled by consensus after a joint review of the article. We specifically analyzed studies which reported iPCAO patients who underwent EVT and a comparison group of iPCAO patients who underwent isolated best medical treatment. Studies that met predefined patient (patients with iPCAO), exposure (EVT), comparison (no EVT), outcome (3-month functional outcome, 3‑month mortality, and sICH) (PECO) criteria were included in double-arm meta-analyses for comparison of matched populations of patients with iPCAO who received and who did not receive EVT.

### Data Extraction

Five authors (A.E., Y.T.S., M.B., A.M., J.P.) extracted key information of the included studies according to a preplanned form and recorded it in a standardized spreadsheet. The following variables were collected: the first author’s name, year of publication, country, study design, number of patients, age, sex, clinical stroke severity depicted by National Institutes of Health Stroke Scale (NIHSS) at baseline and at discharge, occlusion site (P1, P2, P3 segments of PCA), treatment modality (stent-retriever, aspiration thrombectomy, intra-arterial thrombolysis), successful reperfusion (defined as a modified thrombolysis in cerebral infarction score of 2b–3), sICH (as defined by each study), normalization of visual field defect at follow-up, 3‑month functional outcome after stroke depicted by the modified Rankin scale (mRS), and 3‑month mortality. The NIHSS change was defined as baseline NIHSS minus NIHSS at discharge. Functional outcome and mortality were collected preferentially 3 months after stroke, but studies presenting only functional outcome at discharge and in-hospital mortality were also included. Excellent outcome was defined as mRS of 0–1 and favorable outcome was defined as mRS of 0–2. In studies that reported data as median and interquartile range, we converted the values to mean and standard deviation using the method proposed by McGrath et al. [7].

### Quality Assessment

Three authors (A.M., M.B. and H. M. G.) independently assessed the quality of the included studies using the Newcastle-Ottawa quality assessment scale for cohort studies and for case-control studies (NOS star scoring system) [8] and any conflicts were delegated to a fourth author (Y.T.S.). The authors were not blinded to the author names and affiliations. The NOS evaluates three main categories: selection of study population, comparability between study groups and ascertainment of outcome or exposure. The quality of studies scoring 7–9 stars was considered good, 5–6 stars was considered moderate quality and 0–4 stars was considered poor quality.

### Statistical Analysis

We conducted single-arm meta-analyses to estimate mean NIHSS change, and pooled proportions and respective 95% confidence intervals (95% CI) of successful reperfusion, sICH, excellent 3‑month outcome, favorable 3‑month outcome and 3‑month mortality. The meta-analyses for these outcomes were calculated for patients who underwent EVT (with or without IVT). For studies reporting iPCAO patients who underwent EVT and an age-, sex- and NIHSS-matched population of iPCAO patients who received isolated best medical treatment (with or without IVT), we additionally conducted double arm meta-analysis to estimate the difference in mean NIHSS change, and to calculate the pooled risk ratios (RR) for sICH, excellent 3‑month outcome, favorable 3‑month outcome and 3‑month mortality. For the double arm meta-analyses we excluded studies in which all patients who underwent EVT received intra-arterial thrombolysis, to reduce bias related to reperfusion rates and hemorrhagic complications. Because of the presumed heterogeneity between individual study estimates, we performed random effects meta-analyses according to the DerSimonian-Laird method using the “meta” package in R version 4.1.0 (R Project for Statistical Computing, Vienna, Austria). The “metaprop” function was used to calculate pooled proportions, the “metamean” function was used to calculate pooled mean NIHSS changes, and the “metabin” function was used to calculate risk ratios (RR) for binary outcomes in the two groups of patients (EVT versus best medical treatment only). A continuity correction of 0.1 in studies with *n* = 0 of reported outcomes was applied. The inverse variance method was used for study weighting. To exclude bias introduced by study weighting using sampling variance, we also conducted the same double-arm meta-analyses using weighting according to sample size. The I^2^ statistic and Cochrane’s Q‑test were used to assess the heterogeneity among the studies. The I^2^ statistic > 50% indicated presence of substantial heterogeneity and *p*-value < 0.05 indicated statistically significant heterogeneity. We constructed funnel plots for the double-arm meta-analyses to test for publication bias and evaluated plot asymmetry visually. We did not perform Egger’s tests to test funnel plot asymmetry due to the small number of included studies.

## Results

**Fig. 1:**
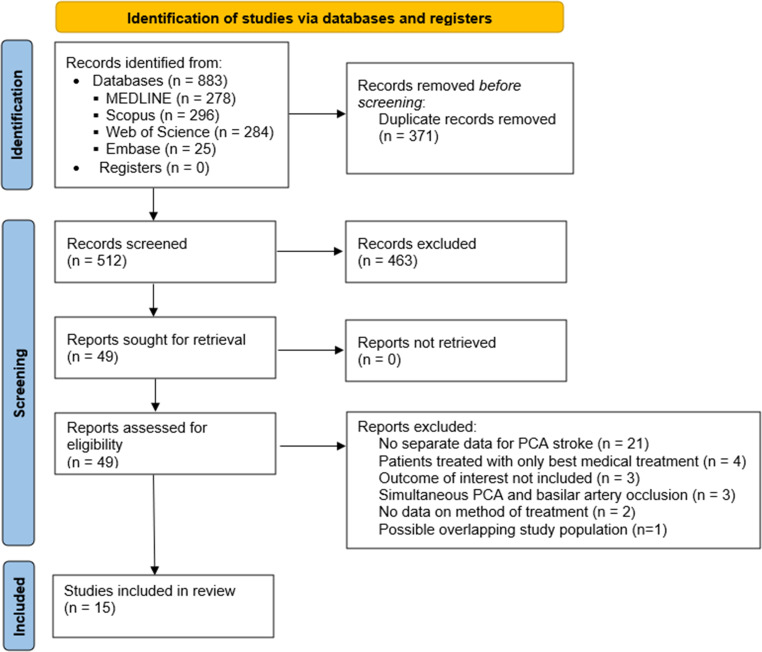
Preferred Reporting Items for Systematic Reviews and Meta-Analyses (PRISMA) flow chart summarizing literature search strategy. *PCA* posterior cerebral artery

The electronic search retrieved 883 articles, of which 371 were duplicate records and were removed before screening (Fig. 1). Among 512 articles which were screened by title and abstract review, 463 articles were excluded for irrelevance. The full text of 49 articles was retrieved for assessment of eligibility, of which 29 articles were excluded because of the following reasons: separate data for patients with PCA occlusion not available (*n* = 21); only patients who underwent best medical treatment only included (*n* = 4); outcomes of interest not included (*n* = 3); patients with simultaneous PCA and other intracranial vessel occlusion (*n* = 3); no information concerning method of treatment (*n* = 2); possible overlapping study population (*n* = 1).

Table 1 summarizes the main characteristics of the included studies (*n* = 15) [9–23]. The majority of the included studies were retrospective cohorts (*n* = 13), one study was a prospective cohort, one study was a retrospective case-control study. The assessment of the quality of the studies using the NOS revealed that the majority of studies (*n* = 10) presented a moderate quality and 4 studies presented a good quality (Supplementary Tables 2 and 3).

**Table 1:** Summary of studies included in the systematic review and meta-analysis

Author	Year	Country	Design	Patients with iPCAO (*n*)	Occlusion site (*n*)	Endovascular treatment						Only best medical treatment				
						Patients (*n*)	Age (years)	Sex (M/F)	Baseline NIHSS	IVT	Endovascular treatment method	Patients (*n*)	Age (years)	Sex (M/F)	Baseline NIHSS	IVT
Brouwer et al. [9]	2022	Netherlands	Multicenter prospective cohort	20	P1 (*n* = 17), P2 (*n* = 2), P3 (*n* = 1)	20	72 (±4.5)	7/13	13 (5–21)	12 (60%)	Stent retriever; aspiration thrombectomy; intra-arterial thrombolysis	–	–	–	–	–
Baik et al. [10]	2022	Republic of Korea	Multicenter retrospective cohort	48	P1 (*n* = 17), P2 (*n* = 31)	48	69.6 (±15.9)	34/14	9 (6–14)	18 (37.5%)	Stent-retriever; aspiration thrombectomy; intra-arterial thrombolysis	–	–	–	–	–
Fischer et al. [11]	2022	Germany	Multicenter retrospective cohort	8	P2 (*n* = 8)	8	NR	NR	NR	NR	Stent retriever	–	–	–	–	–
Baig et al. [12]	2021	USA	Single-center retrospective cohort	21	P1 (*n* = 13), P2 (*n* = 8)	21	71.2 (±10.2)	9/12	9 (5–15)	10 (47.6%)	Stent-retriever; aspiration thrombectomy	–	–	–	–	–
Cunha et al. [13]	2021	Portugal	Single-center retrospective cohort	38	P1 (*n* = 19), P2 (*n* = 16), P3 (*n* = 3)	25	79.3 (±2.4)	16/9	10 (6.0–14.5)	14 (56%)	Stent retriever; aspiration thrombectomy; intra-arterial thrombolysis	13	75.3 (±3.93)	7/6	8 (5.5–10.0)	13 (100%)
Altenbernd et al. [14]	2021	Germany	Single-center retrospective cohort	79	P1 (*n* = 60), P2 (*n* = 19)	79	72.8 (±11.6)	35/44	12 (4–15)	43 (54%)	Stent retriever; aspiration thrombectomy	–	–	–	–	–
Herweh et al. [15]	2021	Germany, USA, Taiwan	Multicenter retrospective cohort	130	P1 (*n* = 62), distal to P1 (*n* = 68)	23	70 (±13.3)	14/9	9 (1–20)^a^	5 (22%)	Mechanical thrombectomy	107	74 (±13.1)	56/51	7 (1–38)^a^	44 (41%)
Meyer et al. [16]	2021	Germany, France, USA, Singapore, Sweden, Spain, Switzerland	Multicenter case-control study	243	P2 (*n* = 199), P3 (*n* = 44)	143	72.66 (±14.1)	79/64	7 (4–11)	57 (40%)	Stent retriever; aspiration thrombectomy; intra-arterial thrombolysis	92	72.2 (±14.3)	42/50	5 (2–10)	53 (58%)
Miszczuk et al. [17]	2021	Germany	Multicenter retrospective cohort	17	P2 (*n* = 14), P3 (*n* = 3)	17	74 (58–81)	NR	10 (5–14)	8 (47%)	Stent retriever; aspiration thrombectomy	–	–	–	–	–
Clarençon et al. [18]	2020	France, Switzerland, USA, Czech Republic, Spain	Multicenter retrospective cohort	22	P1 (*n* = 17), P2 (*n* = 5)	22	66.2 (±14.3)	12/10	14 (8–16)	11 (50%)	Stent retriever	–	–	–	–	–
Memon et al. [19]	2020	USA	Single-center retrospective cohort	15	P1 (*n* = 12), P1/P2 junction (*n* = 3)	15	64 (±17)	6/9	9 (5–15)	7 (46%)	Stent retriever	–	–	–	–	–
Strambo et al. [20]	2020	Switzerland	Single-center retrospective cohort	106	P1 (*n* = 34), P2 (*n* = 72)	21	71 (64–78.2)	8/13	7 (5–8.3)	13 (62%)	Stent retriever; aspiration thrombectomy; intra-arterial thrombolysis	85	76.6 (68.3–83.8)	48/37	7 (4–12)	34 (40%)
Grossberg et al. [21]	2018	USA	Single-center retrospective cohort	3	NR	3	66 (±26.5)	3/0	14 (8–22)	2 (67%)	Stent-retriever; aspiration thrombectomy	–	–	–	–	–
Premat et al. [22]	2018	France	Single-center retrospective cohort	7	P1 (*n* = 3), P2 (*n* = 2), P3 (*n* = 2)	7	NR	NR	7 (5–28)	NR	Stent-retriever; aspiration thrombectomy	–	–	–	–	–
Meier et al. [23]	2011	Switzerland	Single-center prospective cohort	18	P1(*n* = 7), P2 (*n* = 10), P3 (*n* = 1)	9	66 (±11.6)	5/4	9 (6–16)	0	Intra-arterial thrombolysis; mechanical thrombectomy	9	58 (±16.2)	7/2	4 (3–6)	9 (100%)

Values are presented as *n* (%), mean (± SD) or median (IQR)

*NIHSS* National Institutes of Health Stroke Scale, *IVT* intravenous thrombolysis, *M* male, *F* female, *NR* not reported, *iPCAO* isolated posterior cerebral artery occlusion

^a^Median (range)

A total of 461 patients with iPCAO who underwent EVT were reported, 53% of patients were male, mean age was 65 years or older in all included studies but one and median baseline NIHSS ranged between 7 and 14. Among studies which reported site of occlusion (*n* = 458 patients), occlusion of the P1 segment was present in 43% of patients and occlusions distal to P1 were present in 57%. In addition to EVT, 45% of the patients had also been treated with IVT. The majority of studies reporting EVT used stent-retriever, aspiration thrombectomy or combined techniques. Six studies reported patients treated with intra-arterial thrombolysis (*n* = 22 patients). Four studies reported age-, sex- and NIHSS-matched control groups of iPCAO patients who were treated with best medical treatment only (*n* = 297 patients), 48% of whom were treated with IVT. When compared to the group of iPCAO patients who underwent EVT, matched iPCAO patients receiving best medical treatment only less frequently had P1 occlusions (25% versus 43%, *p* < 0.001).

### Outcomes of iPCAO Patients Undergoing EVT

**Fig. 2:**
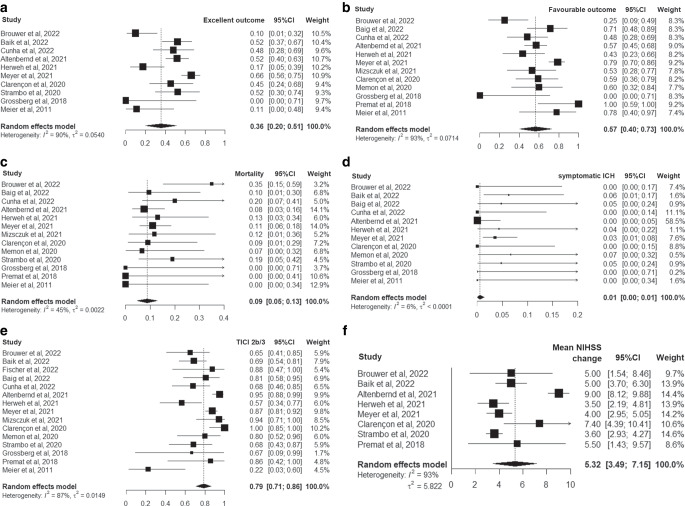
Forest plots of the random effects meta-analysis for outcomes of patients with isolated posterior cerebral artery occlusion who received endovascular therapy: **a** excellent outcome; **b** favourable outcome; **c** mortality; **d** symptomatic intracranial hemorrhage; **e** successful reperfusion; **f** mean National Institutes of Health Stroke Scale change. *ICH* intracranial hemorrhage, *TICI* modified treatment in cerebral ischemia score, *NIHSS* National Institutes of Health Stroke Scale, *95% CI* 95% confidence interval

Fig. 2 summarizes the pooled outcomes of iPCAO patients submitted to EVT. The pooled proportion of patients achieving an excellent 3‑month outcome was 36% (95% CI 20–51%) and of patients achieving a favorable 3‑month outcome was 57% (95% CI 40–73%), while the pooled 3‑month mortality was 8% (95% CI 5–13%). Pooled proportion of sICH was 1% (95% CI 0–1%), pooled successful reperfusion was 79% (95% CI 71–86%), and the pooled mean NIHSS change was 5.3 (95% CI 3.5–7.2). Improvement of visual field defect was only reported in four studies (26/46 patients submitted to EVT experienced normalization of visual field defect) [12, 19, 20, 23], and therefore a meta-analysis was not performed. Significant heterogeneity was found for the meta-analysis of excellent 3‑month outcome (*I*^2^ = 90%, *p* < 0.001), favorable 3‑month outcome (*I*^2^ = 93%, *p* < 0.001), 3‑month mortality (*I*^2^ = 45%, *p* = 0.041) successful reperfusion (*I*^2^ = 87%, *p* < 0.001), and mean NIHSS change (*I*^2^ = 93%, *p* < 0.001).

### Comparison Between iPCAO Patients Receiving EVT Versus Best Medical Treatment only

Among the 15 included studies, there was only one age-, sex- and baseline NIHSS-matched case-control study [16]. For this analysis, we additionally included the cohort studies which reported both iPCAO patients receiving EVT and patients receiving only best medical treatment, in which both groups presented no significant imbalances in age, sex and baseline NIHSS [13, 15, 20]. We excluded the study by Meier et al., as all of the nine included EVT patients were treated with intra-arterial thrombolysis [23]. A total of 434 age-, sex- and baseline NIHSS-matched patients with iPCAO were reported by the four studies included in this analysis (161 patients underwent EVT and 273 patients received best medical treatment only).

**Fig. 3:**
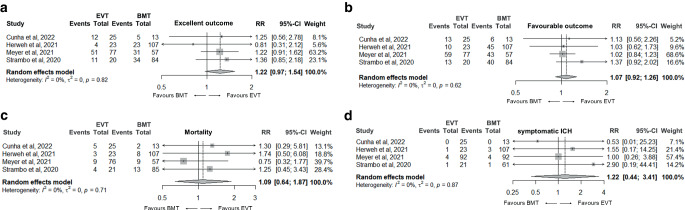
Forest plots of the random effects meta-analysis comparing patients with isolated posterior cerebral artery occlusion who underwent endovascular treatment with age-, sex- and NIHSS-matched patients who received best medical treatment only: **a** excellent outcome; **b** favourable outcome; **c** mortality; **d** symptomatic intracranial hemorrhage. *ICH* intracranial hemorrhage, *EVT* endovascular treatment, *BMT* best medical treatment, *RR* risk ratio, *95% CI* 95% confidence interval

Fig. 3 summarizes the results of the double-arm meta-analyses for the selected outcomes. Both treatment groups did not differ significantly in respect to favorable 3‑month outcome, excellent 3‑month outcome, 3‑month mortality and sICH. Study weighting according to sample size did not change the results significantly. No significant study heterogeneity was found in the four meta-analyses (I^2^ was 0% in the four meta-analyses; Fig. 3). Visual analysis of the funnel plots did not reveal plot asymmetry (Supplemental Fig. 1).

## Discussion

Patients with stroke involving the territory of the PCA are not rare and represent 1 in every 20 acute ischemic stroke cases [3, 4]. Because patients with iPCAO were not included in randomized controlled trials studying the benefit of IVT and/or EVT, acute revascularization treatment decisions in these patients are difficult and should be based on the best available evidence. Our systematic review showed that the currently available evidence on this subject, mainly based on retrospective analyses of consecutive patients, is scarce and that the quality of most studies is not optimal. The main findings of our meta-analysis are the pooled outcomes for iPCAO patients who underwent EVT. About half of the patients with iPCAO treated with EVT presented a favorable 3‑month outcome and 3‑month mortality was low. Although the comparison with the results of the randomized controlled trials of mechanical thrombectomy in anterior circulation large vessel occlusion is not valid because of many methodological reasons and intrinsically different arterial territories, these results appear to be not inferior to those reported by the HERMES collaboration, were in the treatment arm favorable 3‑month outcome was achieved in 46% and mortality was 15% [24]. The pooled successful recanalization proportion in iPCAO patients was 79%, which is relatively high considering that most of the included studies reflect real-life clinical practice, and suggests the feasibility of EVT in this arterial territory. Additionally, the occurrence of sICH was relatively low in comparison with the results from the HERMES collaboration [24].

Unfortunately, systematic information concerning presence of visual field defects at baseline and at follow-up was not available in the majority of studies and therefore could not be analyzed in the current meta-analysis. This is of relevance because iPCAO patients with NIHSS as low as 2 may present significant functional impairment and decrease in quality of life caused by visual problems such as homonymous hemianopsia [25]. Studies of acute reperfusion therapies in this population of patients should therefore include presence of visual field defects in addition to functional outcome and quality of life as a main outcome.

A direct outcome comparison of unmatched iPCAO population of patients may be misleading. Treating physicians in everyday clinical practice probably select patients with more severe neurological deficits and proximal PCA occlusions for EVT, because they must weigh up the severity and functional impact of the neurological deficits and the risks of intervention. Our meta-analysis of studies which included iPCAO patients treated with EVT and age-, sex- and NIHSS-matched IPCAO patients who received only best medical treatment revealed no significant differences in any of the analyzed outcomes (excellent and favorable 3‑month outcome, sICH, and 3‑month mortality). Another recent similar meta-analysis on this subject also found no differences in favorable 3‑month outcome, 3‑month mortality or sICH [26]. This meta-analysis had different search criteria, did not include six relevant studies which we identified in our search [9, 11, 14, 17, 21, 22], therefore including only 338 iPCA patients who underwent EVT (compared to 461 patients in our study), and did not report relevant outcomes such as excellent 3‑month outcome, NIHSS change or improvement of visual field defects. This meta-analysis [26] performed comparisons of EVT versus best medical treatment in the same four studies but used the non-matched patient population of one of the studies [16], which is a potential source of relevant bias. The study by Meyer et al. [16] has the greatest weight in our meta-analyses because of its sampling variance. It is also, however, the study with the largest study population, therefore we found no significant differences in our results when we used sample size for weighting.

These results support the feasibility of EVT in patients with iPCAO and suggest that EVT might not be associated with increased complications leading to sICH and death. We found no significant differences in the outcomes of patients who underwent EVT in comparison to matched patients who received only best medical treatment, but these results must be interpreted with caution. The iPCAO patients treated with EVT included in this meta-analysis were not randomized, and in most cases selected for treatment by the treating physicians, with possible bias introduced by selection of patients with better baseline physiological state, less comorbidities and favorable treatment conditions. Although no suggestion of publication bias was found, this selection bias represents the major limitation of our meta-analysis of non-randomized studies. Despite this, the lack of a significant outcome benefit of EVT compared to best medical treatment in iPCAO patients could be explained by several factors, including lack of statistical power of the current analysis, increased distal embolization during mechanical thrombectomy in patients with posterior circulation occlusions [27], and better recanalization rates of intravenous thrombolysis in medium vessel occlusions when compared to large vessel occlusions [28].

We found significant study heterogeneity in several meta-analyses of outcomes in iPCAO patients who underwent EVT which probably reflect, among others, different treatment selection strategies adopted by the different centers; however, no significant heterogeneity was found for the double-arm meta-analysis of studies comparing both treatment strategies.

We would like to acknowledge several limitations of the current systematic review. As already mentioned, most of the included studies had a retrospective design, which may be responsible for significant within-center heterogeneity in the baseline characteristics of included patients, and lead to missing information at baseline and follow-up. We did not register our study protocol in publicly available international registries for clinical studies or systematic reviews. Systematic information concerning specific neurological deficits (namely presence of visual field defects) and extension of early signs of ischemic in the PCA territory was not available for most studies, both of which are probably robust predictors of short-term outcome and could have decisively influenced the current results. Because of the paucity of data, we could not perform subgroup meta-analyses to study the impact of possible outcome predictors, namely severity of neurological deficits, location of PCA occlusion, IVT and time to treatment. Although two of the included studies provided information on mortality at discharge [17] or 1 month after stroke [22], they represent only 5% of the total included patients, therefore we believe that the risk of bias associated with this limitation is low. Finally, our results demonstrate that published data on acute reperfusion therapy in patients with iPCAO is limited and present only a moderate quality.

## Conclusion

Our systematic review and meta-analysis describes the main short-term to medium-term outcomes of iPCAO patients who undergo EVT. The results suggest the feasibility of EVT in iPCAO, which was associated with low sICH occurrence and low mortality. However, there was no difference in outcomes between the group of patients who underwent EVT and matched patients who received best medical treatment. Whether EVT, at least in selected iPCAO patients, may improve outcome when compared to best medical treatment only awaits clarification in the setting of randomized controlled trials.

## Supplementary Information

Prisma checklist; Newcastle-Ottawa scale for cohort studies and for case-control studies; Funnel plots for the double-arm meta-analyses
